# Impact of health literacy and medication adherence on achieving blood pressure goals among hypertensive patients at the University of Gondar Comprehensive Specialized Referral Hospital, Northwest Ethiopia

**DOI:** 10.1371/journal.pone.0341140

**Published:** 2026-01-20

**Authors:** Alemante Tafese Beyna, Assefa Kebad Mengesha, Habtamu Semagne Ayele, Firomsen Mamuye Dajane, Abreham Honelgn Mule, Assefa Belay Asrie, Tafere Mulaw Belete, Demis Getachew, Tekletsadik Tekleslassie Alemayehu, Gebresilassie Tadesse, Gebremariam Wulie Geremew, Liknaw Workie Limenh, Gizachew Kassahun Bizuneh

**Affiliations:** 1 Department of Pharmacology, School of Pharmacy, College of Medicine and Health Sciences, University of Gondar, Gondar, Ethiopia; 2 Department of Social and Administrative Pharmacy, School of Pharmacy, College of Medicine and Health Sciences, University of Gondar, Gondar, Ethiopia; 3 Department of Psychiatry, School of Medicine, College of Medicine and Health Sciences, University of Gondar, Gondar, Ethiopia; 4 Department of Clinical Pharmacy, School of Pharmacy, College of Medicine and Health Sciences, University of Gondar, Gondar, Ethiopia; 5 Department of Pharmaceutics, School of Pharmacy, College of Medicine and Health Sciences, University of Gondar, Gondar, Ethiopia; 6 Department of Pharmacognosy, School of Pharmacy, College of Medicine and Health Sciences, University of Gondar, Gondar, Ethiopia; Kazan State Medical University: Kazanskij gosudarstvennyj medicinskij universitet Ministerstva zdravoohranenia Rossijskoj Federacii, RUSSIAN FEDERATION

## Abstract

**Background:**

This study assessed hypertensive health literacy and its association with achieving blood pressure goals at the University of Gondar Comprehensive Specialized Hospital, Ethiopia.

**Method:**

A cross-sectional study was conducted with 393 hypertensive patients from September 1 to October 30, 2024, selected through simple random sampling. Data were analyzed using t-tests, one-way ANOVA, and logistic regression, with p < 0.05 considered significant.

**Result:**

Among the 393 participants, only 40.2% of participant achieved their blood pressure goal. Only 15% and 30.8% had high hypertensive health literacy and adherence levels, respectively. Participants living in an urban area (AOR = 5.1, 95% CI: 2.74–9.17, p < 0.001), with higher education (AOR = 2.7, 95% CI: 1.15–6.75, p < 0.023), living with hypertension for more than 10 years (AOR = 3.4, 95% CI: 1.54–7.58, p < 0.002), using three or more antihypertensive drugs (AOR = 0.3, 95% CI: 0.13–0.95, p < 0.041), adhering to treatment (AOR = 2.7, 95% CI: 1.34–5.55, p < 0.005), and having high hypertension health literacy (AOR = 3.8, 95% CI: 1.36–10.90, p < 0.011) were more likely to achieve blood pressure goal. Variables like residence (p < 0.001), marital status (p < 0.004), educational status (p < 0.013), occupation (p < 0.013), number of antihypertensive medications (p < 0.001), and presence of complication (p < 0.002) showed a significantly mean difference in hypertension health literacy score. There is a significant association between hypertensive health literacy and medication adherence with a p-value of < 0.001.

**Conclusion:**

Most participants did not achieve their target blood pressure goals. Hypertensive health literacy and medication adherence were significantly associated with blood pressure goals. To improve these outcomes, health policies should focus on creating community-based educational programs that empower patients with a better understanding of their condition and the importance of following their treatment plans. Additionally, increasing access to healthcare resources and support can provide the guidance patients need to manage their hypertension effectively. By strengthening these initiatives, we can help more individuals take control of their health and achieve better blood pressure management.

## 1. Introduction

### 1.1 Background

Hypertension is a prevalent and serious noncommunicable health condition affecting around 1.28 billion people worldwide, most of them (two-thirds) are living in low and middle-income countries [1]. It contributes to the high burden of premature death worldwide by dramatically increasing the risk of heart disease, atherosclerosis, stroke, kidney failure, and other complications [2,3]. Even with the availability of efficient treatments, only one-third of patients are able to achieve their blood pressure (BP) goal [4,5]. Lowering BP is associated with reducing complications and can significantly lower the risk of stroke, kidney failure, heart disease, and overall mortality [6–8]. Elevated BP is often associated with factors such as poor medication adherence, low health literacy, limited access to healthcare, unhealthy lifestyle habits, and socioeconomic difficulties. [9–11].

Among several factors, Adherence is one of the most important factors in achieving BP goals. Adherence is the degree to which an individual actions such as taking medicine, adhering to a diet, or making lifestyle adjustments align with the advice made by a medical practitioner [12]. Poor adherence to these practices is a major contributor to failing to achieve BP goals, which raises medical expenses, causes unsuccessful treatment modifications, and increases side effects [11]. Furthermore, it has been shown that poor adherence is associated with an increased risk of cardiovascular events and medical costs [13,14]. On the other hand, better medication adherence contributes to lower total medical expenses and helps to achieve BP goals [15]. Adherence to treatment plans helps patients avoid frequent hospital visits, reduces costs, and minimizes medication adjustments and side effects, ensuring better and safer hypertension management [16,17]. To address this, the World Health Organization has released an evidence-based report designed to assist physicians, healthcare managers, and policymakers in enhancing medication adherence worldwide [12]. To assess medication adherence in research settings, various direct and indirect methods have been developed [18,19]. In an outpatient context, four methods have been often employed: pharmacy fill rates, pill counts, electronic monitoring, and self-reporting [20,21]. To improve adherence, it’s important to look at the factors that influence it, and one of the key factors is health literacy [22].

Health literacy refers to a person’s ability to access, understand, and use basic health information and services to make informed decisions about their health [23,24]. It is a critical factor in hypertension management as it directly affects a patient’s ability to adhere to hypertensive medication and achieve targeted BP goals [25]. Patients with low health literacy often struggle to understand medical instructions, like prescription labels and dietary guidelines. This can result in missed doses or taking medications incorrectly [26,27]. This lack of understanding and communication problem can lead to poor medication adherence which results in uncontrolled BP, higher risks of cardiovascular complications, and increased healthcare costs. Studies indicate a strong correlation between medication adherence and health literacy. Individuals with greater health literacy are more likely to adhere to their treatment regimens and have better health results. Therefore, health literacy assessment is required to provide baseline data for creating health education programs and strategies [28].

In Ethiopia, where a significant proportion of the population has limited formal education and healthcare services are often constrained, low health literacy may act as a major barrier to hypertension management. Limited awareness and understanding of hypertension-related information can lead to poor adherence and suboptimal BP control, making this a public health concern [29]. Despite global evidence linking health literacy to medication adherence and BP control, there is a lack of data from Ethiopia. Existing studies in other contexts suggest that improving health literacy can enhance adherence and treatment outcomes [9], yet no research has specifically examined this relationship in Ethiopian hypertensive patients. Addressing this gap, our study provides novel insights into the role of health literacy in hypertension management in Ethiopia. This study aimed to assess hypertension health literacy levels and their association with achieving BP goals among patients with hypertension at the University of Gondar Comprehensive Specialized Hospital (UoGCSH) in Northwest Ethiopia.

## 2. Method

### 2.1 Study design and setting

A cross-sectional study was conducted among hypertension patients at the UoGCSH from September 1 to October 30, 2024. This hospital was founded in 1954 and is located in the Amhara National Regional State’s Central Gondar administrative zone, 750 kilometers northwest of Ethiopia’s capital, Addis Ababa. UoGCSH is the biggest medical facility in northwest Ethiopia, and is essential in the treatment of chronic conditions including hypertension. About 10,000–11,000 hypertensive patients in the region are served by its chronic ambulatory care clinic, which is a vital resource for the treatment of the illness. As the primary referral facility for approximately 12 million people in five zones around the region, UoGCSH is one of the oldest hospitals in Ethiopia with about 950 beds.

### 2.2 Source and study population

All hypertension patients treated at the UoGCSH served as the source population. Specifically, hypertension patients who visited the hospital’s chronic ambulatory care clinic during the data-collecting period served as the study population.

### 2.3 Inclusion and exclusion criteria

Patients who were 18 years of age or older and had been taking antihypertensive drugs for at least three months before the study period were eligible to participate. Patients who suffered from visual or communication difficulties that would have made it difficult for them to participate fully in interviews or finish health literacy surveys or who had cognitive impairments brought on by mental illnesses were excluded from the study.

### 2.4 Sample size calculation and sampling technique

To determine the sample size, the single population proportion formula was applied.


$$n={\left(\text{Z}\;\alpha /\text{2}\right)}^{\text{2}}{\;}^{*}\;\text{P}\;(\text{1}-\text{P})/{\text{d}}^{\text{2}}$$

$$n=(\text{1}.\text{9}{\text{6}}^{*}\text{1}.\text{9}\text{6}{)}^{*}\text{0}.\text{5}\;(\text{1}-\text{0}.\text{5})/\text{0}.\text{0}{\text{5}}^{*}\text{0}.\text{0}\text{5}=\text{3}.\text{8}{\text{4}}^{*}\text{0}.{\text{5}}^{*}\text{0}.\text{5}/\;\text{0}.\text{0}\text{0}\text{2}\text{5}=\text{3}\text{8}\text{4}$$

Where, p = Proportion in the target population with sufficient health literacy. Since no previous research has been done in Ethiopia, there is no realistic estimate, thus we choose to maximize the sample size by using 50%, or 0.5., n = calculated sample size, (α = 0.05), 95% confidence interval (Z α/2 = 1.96), and absolute precision or margin of error, 5% (d = 0.05). The total sample size for hypertension patients was 384. By adding a 10% non-response rate of 384*10% = 38.4, the final sample size was 423.

Participants were selected using a simple random sampling method. We created a sampling frame from patient registration records at the University of Gondar Comprehensive Specialized Referral Hospital during the study period. Data collection occurred on hypertension follow-up days, specifically on Wednesdays, for two months, from September 1 to October 30, 2024. On each follow-up day, approximately 53 patients were chosen. Each eligible patient was assigned a unique identifier, and a random number generator was utilized to ensure that every individual had an equal chance of being included.

### 2.5 Data collection tools and procedures

Data were collected using a standardized validate questionnaire adapted from previous studies [30–32]. The data were collected using an interview-based method by two final-year pharmacy students. Before starting the data collection process, the study’s objectives were briefed to participants and their written consent was gained to participate. The data collection tool’s first portion contains demographics and clinical information, the subsequent sections evaluate participant’s hypertensive health literacy level, and Adherence towards hypertensive medications. The demographic factors encompassed age, sex, residence, occupation, educational status, income, duration of hypertension, and complications. Participant’s hypertensive health literacy level was assessed using a 15-item comprehensive question. The question item was designed based on insights from previous studies. Participants had a 5-Likert scale of possible answers to choose from. Which included options ranging from: “strongly disagree,” “disagree,” “neutral,” “agree,” and “strongly agree.” Each hypertensive health literacy question received a score of 1 for “strongly disagree,” 2 for “disagree,” 3 for “neutral,” 4 for “agree,” and 5 for “strongly agree.” The overall hypertensive health literacy score, ranging from 15 to 75, was determined by summing up the individual item scores. Hypertensive health literacy was classified as “high” for scores of 57 and above, “medium” for scores between 45 and 56, and low for scores of 44 and below [31]. The adherence assessment tool used to evaluate the level of medication adherence among hypertensive patients consisted of 6-item questions. Participants responded using a 2-point Likert scale, with options ranging from “No” to “Yes.” Each question received a score of 0 for “No” and 1 for “Yes,” resulting in an overall adherence score that ranged from 0 to 6, calculated by summing the individual item scores. Adherence was categorized as high (score = 0), medium (scores of 1–2), and low (score > 2) [32]. To facilitate interpretation, all medication adherence scores were converted to a 0–100 scale, with a threshold of 80% applied to classify patients as “adherent” (scores of ≤1) or “non-adherent” (scores of ≥2), a cutoff selected based on previous study [33].

### 2.6 Data quality control

The questions were initially translated into Amharic and then back-translated into English to ensure their original meaning was preserved. This process was conducted to maintain clarity, and accuracy, and to reduce any potential biases in the responses. Intensive training was given to allocate data collectors and supervisors. The training covered data collection procedures, study goals, and the clarity of terminology and tools. It also emphasized the importance of promptly organizing and submitting the gathered data. To ensure that the questions and data collection tool were consistent, 5% (22) of the participants who were excluded from the final study were pretested. Rewording and refining the data collection tool were among the adjustments made based on the findings. Throughout the data collection process, the supervisor and the lead investigator conducted daily follow-ups. The lead investigators and the supervisor verified each questionnaire’s daily completeness. The health literacy measurement tool used in this study consists of a 15-item questionnaire tailored to assess the health literacy of hypertensive patients. Modifications included adjusting questions to specifically address hypertension-related topics, such as understanding hypertension management, interpreting blood pressure levels, and dietary considerations related to sodium intake. Following these adaptations, the Cronbach’s alpha for the tool was found to be 0.836, indicating acceptable reliability for the target population.

### 2.7 Data entry and statistical analysis

The data were analyzed using SPSS version 25.0 and presented as frequencies and percentages through descriptive texts, tables, and figures. The relationship between sociod-demographic factors, clinical data, adherence level, hypertension-related health literacy level, and BP goal was evaluated using bivariate and multivariate logistic regression. Variables with a p-value less than 0.25 in the bivariate analysis were included in multivariate logistic regression models. The one-way ANOVA test and the independent samples t-test were used to evaluate the mean hypertensive health literacy score differences across the various groups. The relationship between patients’ levels of adherence and hypertensive health literacy was assesd using a chi-square test. A 95% confidence interval and a P-value < 0.05 were considered statistically significant.

### 2.8 Operational definition

**High hypertensive health literacy:** If a hypertensive health literacy score is 75% and above (score 57 and above) [30,31]

**Medium hypertensive health literacy:** If a hypertensive health literacy score is 60–74% (score 45–56) [30,31]

**Low hypertensive health literacy:** If a hypertensive health literacy score is less than 59% (score 44 and below) [30,31]

**Adherent:** If a hypertensive adherence score is ≤ 1 [32,33]

**Non-adherent:** If a hypertensive adherence score is ≥ 2 [32,33]

**Achieved BP goal:** Based on JNC 8 guidelines, this refers to maintaining a BP below 140/90 mmHg for most adults or below 150/90 mmHg for individuals aged 60 and older without diabetes or chronic kidney disease [34].

## 3. Result

### 3.1 Demographic characteristics and clinical information

Of the 423 participants, 393 completed the questionnaire, resulting in a response rate of 92.9%. Half of participants, 199 (50.6%), were aged between 40–60 years. Nearly half of the participants, 202 (51.4%), were male and 205 (52.2%) live in rural areas. A significant proportion, 115 (29.3%), had a hypertension duration of 3–5 years, 273 (69.5%) experienced complications, 225 (57.3%) had no family history of hypertension, and 198 (50.4%) were taking two antihypertensive medications. The full dataset is provided as S1 Dataset (Table 1).

**Table 1:** Socio-demographic characteristics and clinical information of hypertension patients at UoGCSH, Ethiopia, 2024 (N = 393).

Variable		Frequency (%)
Age	<40	80 (20.4)
	40-60	199 (50.6)
	>60	114 (29.0)
Gender	Female	191 (48.6)
	Male	202 (51.4)
Residence	Rural	205 (52.2)
	Urban	188 (47.8)
Marital status	Divorced/widowed	141 (35.9)
	Married	170 (43.3)
	Single	82 (20.9)
Educational status	Unable to read and write	97 (24.7)
	Able to read and write	64 (16.3)
	Elementary school	76 (19.3)
	High school	71 (18.1)
	Higher institute	85 (21.6)
Monthly income	<3000 birr	131 (33.3)
	3000-7000 birr	120 (30.5)
	7000-12000 birr	91 (23.2)
	>12000 birr	51 (13.0)
Occupation	Farmer	85 (21.6)
	Government employ	136 (34.6)
	House wife	44 (11.2)
	Merchant	32 (8.1)
	Retire	46 (11.7)
	Other	50 (12.7)
Duration of hypertension	<3 year	111 (28.2)
	3-5 year	115 (29.3)
	5-10 year	86 (21.9)
	>10 year	81 (20.6)
Family history of hypertension	No	225 (57.3)
	Yes	168 (42.7)
Number of antihypertensive medications	One	50 (12.7)
	two	198 (50.4)
	3 and more	145 (36.9)
Complication	No	120 (30.5)
	Yes	273 (69.5)

### 3.2 Hypertension health literacy of the respondents

**Fig 1:**
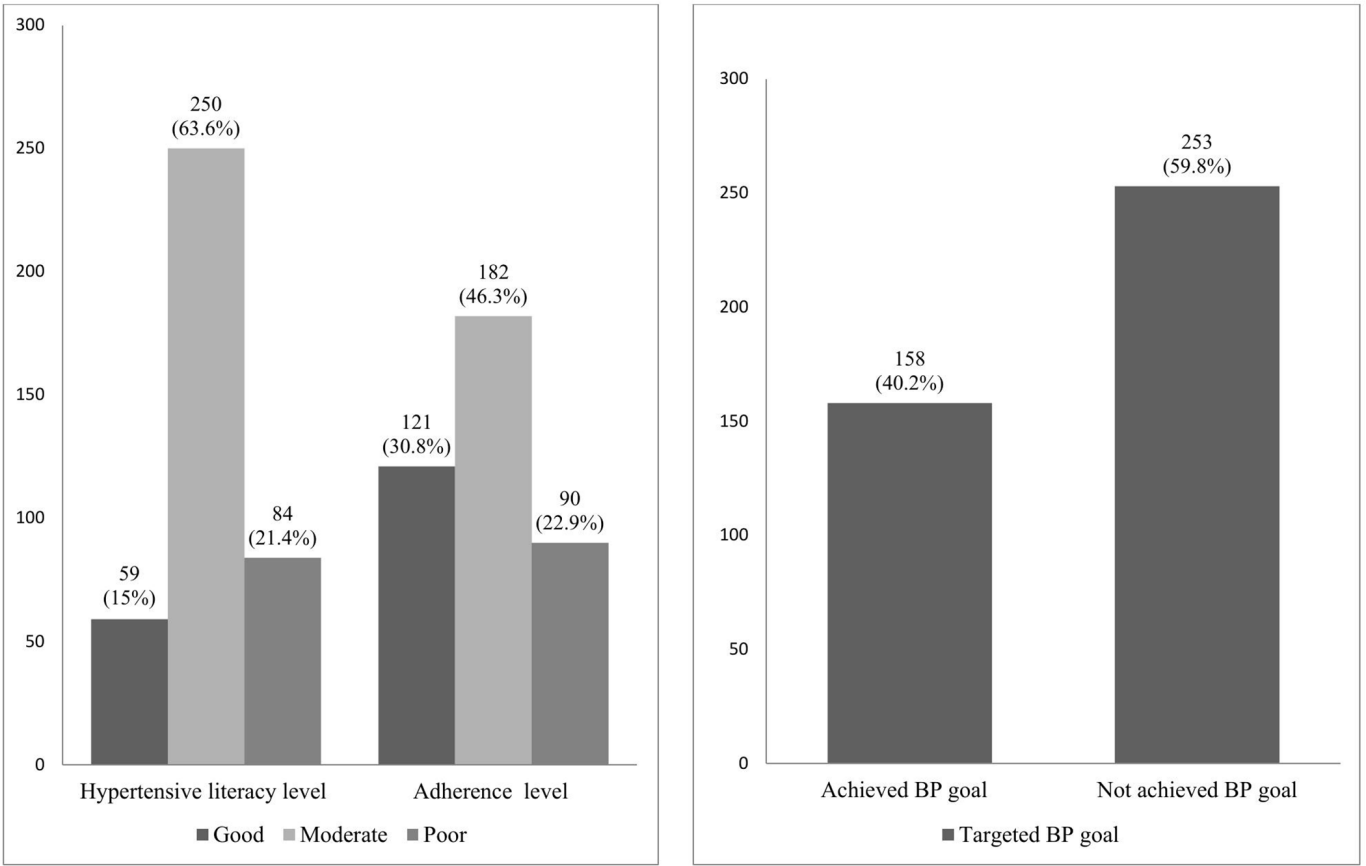
Description of hypertension health literacy, adherence levels, and BP goal achievement among hypertensive patients at UoGCSH, Ethiopia, 2024 (N = 393).

Among hypertension patient participants, the majority 250 (63.6%) of participants showed a medium hypertension literacy level (Fig 1). According to the hypertension literacy evaluation, the majority, 169 (43.0%) of respondents strongly agreed that they could read and understand educational materials and booklets. Furthermore, most of the participants, 343 (87.3%), either agree or strongly agree that they know and practice the appropriate storage conditions of hypertension medications. In addition to this, almost half of the participants 198 (50.4%), agree that they determine the table salt (NaCl_2_) content per serving from the nutrition label. While only a few participants, 35 (8.9%) agreed or 9 (2.3%), strongly agreed that they could understand information about hypertension when presented as probabilities, ratios, or graphs (Table 2).

**Table 2:** Description of the hypertension health literacy responses of hypertension patient at UoGCSH, Ethiopia, 2024 (N = 393).

		Strongly disagree (%)	Disagree (%)	Neutral (%)	Agree (%)	Strongly agree (%)
1	Read and understand educational materials and booklets	19 (4.8)	135 (34.4)	20 (5.1)	50 (12.7)	169 (43.0)
2.	Understand the written information provided at the appointment	40 (10.2)	150 (38.2)	20 (5.1)	164 (41.7)	19 (4.8)
3.	Comprehend the information I sought on hypertension	10 (2.5)	5 (1.3)	35 (8.9)	321 (81.7)	22 (5.6)
4.	Understand the information on hypertension management from the health-care provider	20 (5.1)	15 (3.8)	47 (12.0)	275 (70.0)	36 (9.2)
5.	Judge if hypertension-related information is reliable	22 (5.6)	21 (5.3)	54 (13.7)	235 (59.8)	61 (15.5)
6.	Alter the appointment date or time for a medical checkup	13 (3.3)	186 (47.3)	70 (17.8)	119 (30.3)	5 (1.3)
7.	Calculate the next time to take hypertension medication	9 (2.3)	40 (10.2)	35 (8.9)	292 (74.3)	17 (4.3)
8.	Determine the table salt (NaCl_2_) content per serving from the nutrition label	39 (9.9)	94 (23.9)	40 (10.2)	198 (50.4)	22 (5.6)
9.	Interpret if my blood pressure level is within the normal range	15 (3.8)	108 (27.5)	35 (8.9)	218 (55.5)	17 (4.3)
10.	Understand information on hypertension presented as probabilities, ratios, or on graphs	30 (7.5)	264 (67.2)	55 (14.0)	35 (8.9)	9 (2.3)
11.	Ask health professionals a question	71 (18.1)	297 (75.6)	10 (2.5)	5 (1.3)	10 (2.5)
12.	Explain my hypertension condition to a healthcare provider	23 (5.9)	81 (20.6)	19 (4.8)	265 (67.4)	5 (1.3)
13.	Convey the reason why I should have a control table salt (NaCl_2_) in my diet	44 (11.2)	69 (17.6)	35 (8.9)	218 (55.5)	27 (6.9)
14.	Knowing and practicing the appropriate storage conditions of hypertension medications	5 (1.3)	5 (1.3)	40 (10.2)	297 (75.6)	46 (11.7)
15.	Understand all hypertension-related medication information	10 (2.5)	15 (3.8)	75 (19.1)	242 (61.6)	51 (13.0)

### 3.3 Mean score of hypertension health literacy

The mean hypertensive health literacy score among the respondents was calculated as 49.9, with a standard deviation of 6.1, indicating variability. The independent samples t-test and one-way ANOVA revealed a significantly higher mean hypertension health literacy among participants living in urban areas (p < 0.001), those who are single (p < 0.004), participants with a high school level of education (p < 0.013), and those taking three or more medications (p < 0.001) (Table 3).

**Table 3:** Independent samples t-test and one-way ANOVA for mean hypertension health literacy score of the hypertension patients at UoGCSH, Ethiopia 2024 (N = 393).

Variable		Mean ±SD	F	Sig (P-value)
Residence	Rural	49.53 ± 6.82	17.62	0.001^*^
	Urban	50.44 ± 5.15		
Marital status	Divorced/widowed	48.84 ± 5.01	5.63	0.004^*^
	Married	50.10 ± 6.60		
	Single	51.63 ± 6.31		
Educational status	Unable to read and write	48.69 ± 7.28	3.23	0.013^*^
	Able to read and write	49.81 ± 3.90		
	Elementary school	49.57 ± 7.43		
	High school	52.00 ± 5.05		
	Higher institute	50.20 ± 4.97		
Occupation	Farmer	48.76 ± 7.06	2.93	0.013*
	Government employ	50.79 ± 5.69		
	House wife	48.72 ± 7.51		
	Merchant	48.21 ± 6.30		
	Retire	51.71 ± 4.05		
	Other	50.38 ± 4.60		
Number of antihypertensive medications	One	51.00 ± 5.94	8.44	0.001*
	two	48.74 ± 6.25		
	3 and more	51.28 ± 5.59		
Complication	No	48.54 ± 5.82	9.70	0.002*
	Yes	50.59 ± 6.10		

### 3.4 Adherence level of the respondent

**Fig 2:**
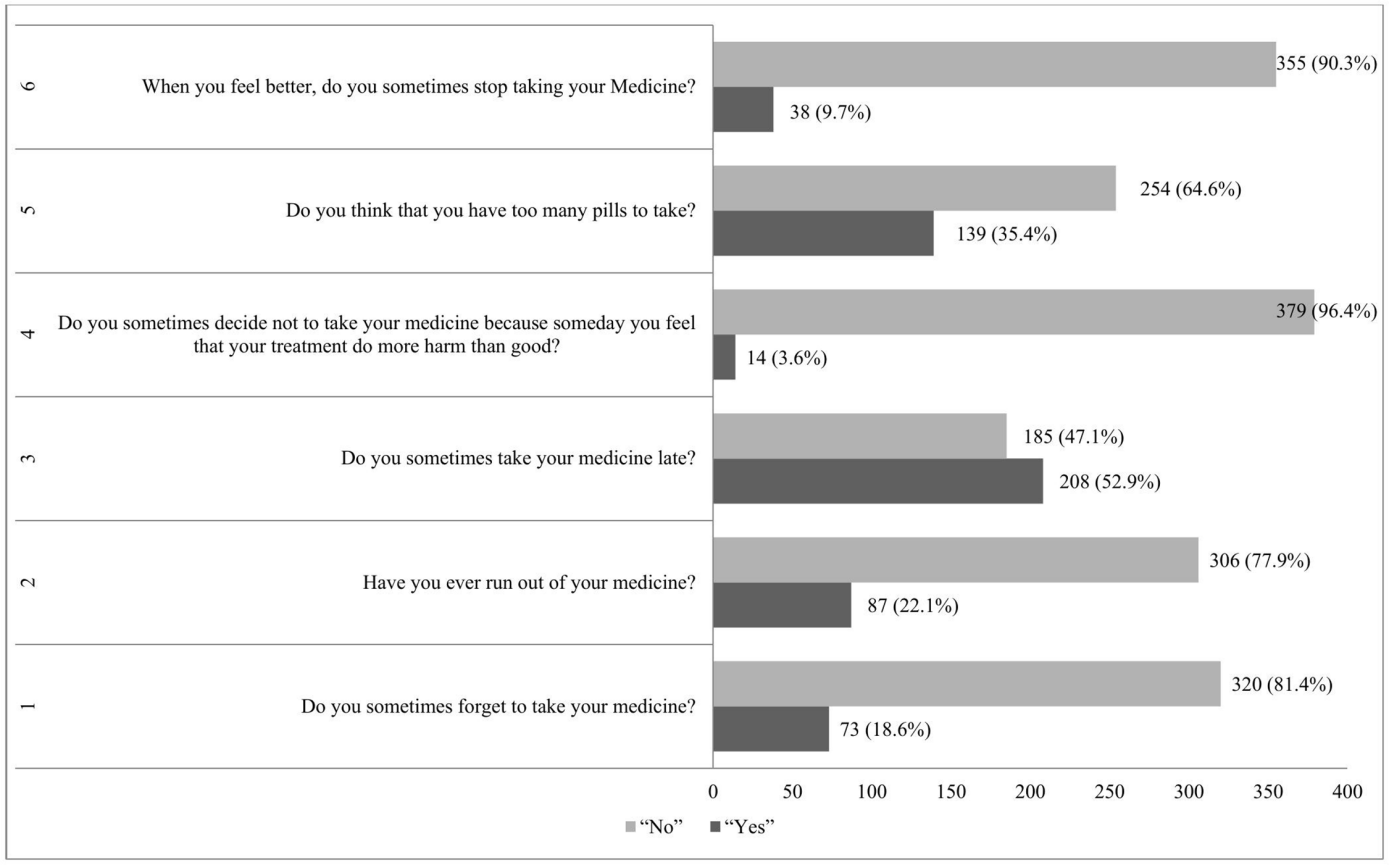
Description of the medication adherence responses of hypertension patient at UoGCSH, Ethiopia, 2024 (N = 393).

Among the 393 participants, 121 (30.8%) showed high adherence, and 182 (46.3%) had medium adherence, with a mean adherence score of 1.43 (SD ± 1.35) out of 6. Fig 2 show that, nearly half of participants, 208 (52.9%) acknowledged sometimes they take their medications later. Furthermore, 139 (35.4%) individuals said that the quantity of medicines they had to take was overwhelming them. However, the majority of participants continued to take their medicine even after their symptoms improved, as evidenced by the fact that just a tiny percentage 38 (9.7%) (38, 9.7%) reported stopping it after they felt better (Fig 2).

### 3.5 Association between hypertension health literacy and adherence profiles

Among the participants with low hypertensive literacy, the majority respondents, 44 (52.4%) had low adherence to their treatment. In contrast, those with medium hypertensive literacy, 137 (53.8%), showed a higher rate of medium adherence. While a smaller percentage, 38 (15.2%) demonstrated low adherence. As the Pearson Chi-square test shows, the difference in hypertensive health literacy levels between these groups was statistically significant, with a p-value of < 0.001. This shows that hypertensive literacy is a significant factor influencing adherence levels. (Table 4)

**Table 4:** Cross-tabulation and Pearson Chi-square test of hypertension health literacy level and adherence of the hypertension patients at UoGCSH, Ethiopia, 2024 (N = 393).

	Adherence level			Total	p-value
	Low	Medium	High		
Low hypertensive literacy	44 (52.4%)	15 (17.9%)	25 (29.8%)	84	< 0.001^*^
Medium hypertensive literacy	38 (15.2%)	137 (54.8)	75 (30.0%)	250	
High hypertensive literacy	8 (13.6)	30 (50.8)	21 (35.6)	59	
Total	90	182	121	393	

### 3.6 Factors associated with achieving BP goal

Based on our findings several important variables are significantly associated with achieving the desired BP goal. Hypertension patient living in an urban area was significantly associated with the likelihood of achieving their BP goal. This indicates that living in an urban area was 5.1 times more likely to achieve their BP goal (AOR = 5.1, 95% CI: 2.74–9.17, p = 0.001) than those participants living in rural areas. Participants with higher education were 2.7 times more likely to achieve their BP goal (AOR = 2.7, 95% CI: 1.15–6.75, p = 0.023) than those participants who were unable to write and read. Similarly, those who had been hypertensive for more than 10 years were 3.4 times more likely to achieve their BP goal (AOR = 3.4, 95% CI: 1.54–7.58, p = 0.002) compared to those with hypertension duration of less than three years. Another important-significantly associated factor was the number of antihypertensive drugs taken. Compared to people taking a single antihypertensive drug, those taking three or more had a 0.3 times higher chance of achieving their BP goal (AOR = 0.3, 95% CI: 0.13–0.95, p = 0.041). Medication adherence was another associated significant factor, adherent hypertension patients are 2.7 times more likely to achieve their BP goal (AOR = 2.7, 95% CI: 1.34–5.55, p = 0.005) compared to non-adherent hypertension patients. Finally, hypertension health literacy also played a crucial role, hypertension patients having high hypertension health literacy were 3.8 times more likely to achieve their BP goal (AOR = 3.8, 95% CI: 1.36–10.90, p = 0.011) (Table 5).

**Table 5:** Multivariable logistic regression analysis on factors associated with achieving BP goal among hypertensive patients at UoGCSH, Ethiopia, 2024 (N = 393).

Variable		Targeted BP goal (Less than 140/90 mmHg)		COR (95% CI)	AOR (95% CI)	p-value
		Achieved (%)	Not Achieved (%)			
Age	<40	40 (50)	40 (50)	1	1	
	40-60	71 (35.7)	128 (64.3)	0.5 (0.32-0.93)	0.7 (0.34-1.63)	0.478
	>60	47 (41.2)	67 (58.8)	0.7 (0.39-1.24)	0.7 (0.31-1.62)	0.418
Residence	Rural	46 (22.4)	159 (77.6)	1	1	
	Urban	112 (59.6)	76 (40.4)	5.1 (3.28-7.89)	5.1 (2.74-9.17)	0.001^*^
Marital status	Divorced/widowed	57 (40.4)	84 (59.6)	1	1	
	Married	75 (44.1)	95 (55.9)	1.1 (0.74-1.82)	0.9 (0.45-1.81)	0.775
	Single	26 (31.7)	56 (68.3)	0.6 (0.38-1.21)	0.5 (0.23-1.38)	0.215
Educational status	Unable to read and write	25 (25.8)	72 (74.2)	1	1	
	Able to read and write	15 (23.4)	49 (76.6)	0.8 (0.42-1.84)	0.4 (0.16-1.12)	0.087
	Elementary school	26 (34.2)	50 (65.8)	1.4 (0.77-2.88)	1.8 (0.76-4.25)	0.176
	High school	36 (50.7)	35 (49.3)	2.9 (1.54-5.67)	1.3 (0.55-3.38)	0.498
	Higher institute	56 (65.9)	29 (34.1)	5.5 (2.93-10.53)	2.7 (1.15-6.75)	0.023^*^
Monthly income	<3000 birr	43 (32.8)	88 (67.2)	1	1	
	3000-7000 birr	46 (38.3)	74 (61.7)	1.2 (0.75-2.13)	1.1 (0.49-2.28)	0.883
	7000-12000 birr	39 (42.9)	52 (57.1)	1.5 (0.88-2.66)	1.2 (0.61-2.68)	0.498
	>12000 birr	30 (58.8)	21 (41.2)	2.9 (1.50-5.69)	1.3 (0.56-3.39)	0.472
Occupation	Farmer	30 (35.3)	55 (64.7)	1	1	1
	Government employ	90 (66.2)	46 (33.8)	3.5 (2.03-6.33)	1.8 (0.88-3.86)	0.100
	House wife	6 (13.6)	38 (86.4)	0.2 (0.11-0.76)	0.5 (0.16-1.59)	0.248
	Merchant	6 (18.8)	26 (81.2)	0.4 (0.15-1.14)	0.3 (0.09-1.08)	0.067
	Retire	11 (23.9)	35 (76.1)	0.5 (0.25-1.29)	0.4 (0.14-1.12)	0.083
	Other	15 (30.0)	35 (70.0)	0.7 (0.37-1.66)	0.4 (0.16-1.16)	0.97
Duration of hypertension	<3 year	36 (32.4)	75 (67.6)	1	1	
	3-5 year	41 (35.4)	74 (64.3)	1.1 (0.66-2.01)	0.5 (0.23-1.13)	0.099
	5-10 year	35 (40.7)	51 (59.3)	1.4 (0.79-2.56)	1.4 (0.67-3.22)	0.336
	>10 year	46 (56.8)	35 (43.2)	2.7 (1.51-4.95)	3.4 (1.54-7.58)	0.002^*^
Number of antihypertensive medications	One	23 (46.0)	27 (54.0)	1	1	
	two	85 (42.9)	113 (57.1)	0.8 (0.47-1.54)	0.7 (0.31-2.01)	0.621
	3 and more	50 (34.5)	95 (65.5)	0.6 (0.32-1.18)	0.3 (0.13-0.95)	0.041^*^
Adherence level	Non-adherent	47 (27.6)	123 (72.4)	1	1	
	Adherent	111 (49.8)	112 (50.2)	2.5 (1.69-3.97)	2.7 (1.34-5.55)	0.005^*^
Hypertensive health literacy level	low	21 (25.0)	63 (75.0)	1	1	
	Medium	109 (43.6)	141 (56.4)	2.3 (1.33-4.03)	2.1 (0.95-4.79)	0.064
	High	28 (47.5)	31 (52.5)	2.7 (1.33-5.51)	3.8 (1.36-10.90)	0.011^*^

* Significant at p < 0.05.

## 4. Discussion

In the present study, 158 (40.2%) of participants had achieved the targeted BP goal. This is in line with the study findings done in South Asia (42%) [35], Ghana (42.3%) [36], Somalia (38.3) [37], China (44.6%) [38], Kenya (40%) [39], Thailand (46.6) [40], Iran (44.9) [41], and Dessie City (44.2) [42]. However, this is higher than the findings reported from Congo (22.5%) [43], South Africa (24.5%) [44], Nigeria (35%) [45], Panama (33.3%) [46], and Zimbabwe (32.8%) [47]. Nevertheless, our study finding is lower than the study conducted in Israel (64.1%) [48]. The reason for this discrepancy might be due to differences in health care policy and access to essential medications can significantly affect BP control. Higher percentages of achieving BP goals are commonly associated with nations with advanced healthcare systems because they frequently have better monitoring, more efficient treatment plans, and better medication supply to patients [49–51]. The other possible reason might be also the variations in health literacy and medication adherence levels between the countries. People with better health literacy are more likely to understand the treatment plan provided by health professional and adhere to medication; this helps them to achieve targeted BP goals [52,53]. On the other hand, people with lower health literacy and poor medication adherence may face challenges in managing their condition effectively, contributing to health disparities in certain populations [9,14]. Other possible for the disparity in the study outcomes might be due to variations in the cut-off points or differences in JNC classifications used to define achieved BP goals across the studies [54]. Furthermore, this discrepancy may be due to variations in the socio-demographic and behavioral characteristics as well as variations in the study environments.

Our study shows a significant association (p < 0.001) between hypertension health literacy and medication adherence. Individuals with higher hypertensive health literacy showed better medication adherence. These findings are consistent with other studies showing that health literacy directly impacts medication adherence [55,56]. The reason for this may be that people with high health literacy are more likely to understand their condition, the importance of their treatment, and how to take their medications properly. This understanding makes it easier for them to follow their treatment plans consistently [57]. Conversely, those with low health literacy frequently find it difficult to completely comprehend the significance of their prescription, how to take it properly, and when to take it. This can lead to missed doses or incorrect use. Additionally, they may also feel less confident asking their healthcare providers for clarification or support [55]. However, it is important to consider that this relationship may be influenced by various confounders [58]. Factors such as age, education level, and socioeconomic status may play a significant role in both health literacy and medication adherence [59]. Individuals with higher education may have better reading and comprehension skills, enabling them to understand medical instructions more effectively, while those with lower education may struggle with complex health information [60]. Similarly, older adults might experience cognitive decline or difficulty processing new medical information, affecting both their literacy and adherence. Socioeconomic status also plays a role, as individuals with higher income and better access to healthcare services may have greater exposure to health education and resources, facilitating better adherence. Although our findings suggest a strong link between health literacy and medication adherence, adjusting for these confounders in future studies using multivariate analysis will be essential to determine the independent effect of health literacy. Without such adjustments, the observed association may partially reflect the influence of these other factors rather than a direct causal relationship However, our findings are inconsistent with some other studies that have reported no association between health literacy and medication adherence [61]. This inconsistency highlights the need for further research to better understand the relationship and identify other factors that might play a role in influencing medication adherence.

Additionally, this study identified factors significantly associated with achieving the targeted BP goal through both bivariate and multivariate analyses. Participant hypertensive patients living in urban areas were 5.1 times more likely to achieve their BP goals compared to those living in rural areas. This might be due to those participants who live in urban areas having better access to health facilities and essential medication that helps them to achieve their target BP goal. They may also benefit from great exposure to media that give them access to health education, and awareness campaigns, enabling them to better understand and manage their condition [62]. Additionally, there are usually more medical specialists in urban areas who can offer advice and assistance. On the other hand, rural individuals can encounter obstacles such as poor health literacy, transportation issues, and restricted access to healthcare, which could make it more difficult to effectively treat hypertension [63]. Our research finding is aligns with the result from a systematic review of urban-rural hypertension differences in low- and middle-income countries (1990–2020), which noted a stronger increase in prevalence in rural areas [64]. Additional important variables significantly associated with achieving the desired BP goal are the quantity of antihypertensive drugs taken and educational attainment. Participant’s complete higher educational institutions were more likely to achieve the targeted BP goals, whereas those on three or more hypertensive drugs were negatively associated with targeted BP goal achievement. The reason for this might be due to participant’s complete higher education institutions more aware of the importance of staying healthy and are more likely to follow their treatment plans. They often maintain a healthy lifestyle by adopting well-informed lifestyle habits and attending routine checkups. This demonstrates how education can improve health and why it’s critical to encourage health education for all people, particularly those who have less access to it [65]. On the other hand, taking three or more medications negatively impacts BP management, the reason might be that complexity of multiple medications can reduce adherence and complicate treatment. Our result is consistent with studies showing that polypharmacy often leads to lower adherence and worse outcomes [66]. Other important variables significantly associated with achieving the desired BP goal are a high level of hypertensive health literacy and medication adherence. Participants with high levels of health literacy and who adhere to their medication were more likely to achieve the targeted BP goals. The reason for this might be due Participant with a high level of hypertensive health literacy understand their hypertension status and fully aware of the importance of achieving targeted BP goals and how lifestyle changes can help. This knowledge allows them to make smarter choices about their health. Simultaneously, adhering to their medication ensures their treatment works as intended and helping to keep their BP stable. Together, these factors make it much easier to achieve and maintain the target BP. This result is consistent with studies showing that health literacy and adherence are associated with BP goals [52,53].

The analysis using independent samples t-test and one-way ANOVA showed that people living in urban areas and those who are single had higher mean hypertension health literacy scores. This may be because hypertensive patients who live in urban areas have easier access to resources, information, and healthcare that aid in the understanding and treatment of hypertension. For single individuals, they may have more time and attention to learn about their health and make better judgments about how to manage their illness if they have fewer household responsibilities [62,67]. In addition to this, participants with health complications, those taking three or more medications, retirees, and individuals who completed high school had higher mean hypertension health literacy scores. This might be the case because individuals who are managing several medications or health conditions tend to be more interested in their care, attempting to better understand their condition and adhere to treatment regimens. People who are retired may have more time to concentrate on their health and educate themselves about their condition because they have fewer work-related obligations. Likewise, those with more education tend to be better able to comprehend medical information, which helps them make wise choices regarding the treatment of their hypertension. These factors suggest that increased engagement, time, and education play a key role in improving health literacy among individuals with hypertension [68,69].

Improving health literacy and medication adherence requires practical, patient-centered approaches. Community programs like educational workshops, mobile health (mHealth) reminders, and peer support groups can help raise awareness and encourage better management of hypertension [70]. One-on-one counseling by pharmacists should also be a key part of routine care, giving patients clear, personalized guidance on their medications and lifestyle changes. Simplifying medication instructions and involving family members in the process can make a big difference. Strengthening these strategies in both clinical practice and policy can help more patients achieve better BP control and overall health [71].

This study aims to explore the relationship between hypertensive health literacy, blood pressure control status, and medication adherence. However, one important limitation is that our cross-sectional design only provides a snapshot of data at a single point in time, making it difficult to draw causal conclusions about these relationships. Additionally, self-report bias could impact our adherence measures, as patients may tend to over report their medication use to appear more compliant. There’s also the issue of generalizability, since our sample comes from a single hospital, which may not reflect the broader population. To gain a deeper understanding of these connections, future research using cohort or case study designs would be more informative.

## 5. Conclusion

This study showed that most participants struggled to achieve their target BP goals. Only 15% had a high level of hypertensive health literacy, and 30.8% were strongly adhering to their medication plans. We found a significant association between hypertensive health literacy and medication adherence with targeted BP goal, highlighting the crucial role of educating patients about their condition. These findings underline the need for tailored strategies to improve health literacy and medication adherence, which could help more patients reach their BP goals. Healthcare providers should engage in personalized conversations with patients, offering clear guidance and support. Regular follow-up visits can help patients stay on track with their treatment. Community-based educational initiatives and collaborative efforts between healthcare workers and patients are key to improving BP control and overall well-being.

## Supporting information

S1 Dataset Comprehensive dataset of hypertensive patients. (XLSX)
